# Effectiveness of Organizational Interventions to Reduce Burnout in the Workplace: A Systematic Review

**DOI:** 10.3390/ijerph23050556

**Published:** 2026-04-25

**Authors:** Diana Araújo, Ana Bártolo, Cláudia Fernandes, Anabela Pereira, Sara Monteiro

**Affiliations:** 1 Department of Education and Psychology, University of Aveiro, 3810-193 Aveiro, Portugal; 2 RISE-HEALTH@Upt, Portucalense University, 4200-072 Porto, Portugal; ana.bartolo@upt.pt; 3 CATIM—Portuguese Technological Center for the Metalworking Industry, 4100-414 Porto, Portugal; cjsfernandes@gmail.com; 4 Department of Psychology, University of Évora, 7002-554 Évora, Portugal; anabela.pereira@uevora.pt; 5 Center for Research in Education and Psychology (CIEP), University of Évora, 7002-554 Évora, Portugal; 6 CINTESIS@RISE, Department of Education and Psychology, Campus Universitário de Santiago, University of Aveiro, 3810-193 Aveiro, Portugal; smonteiro@ua.pt; 7 Department of Social Sciences and Management, Open University, 1269-001 Lisbon, Portugal; 8 Center for Global Studies, Open University, 1269-001 Lisbon, Portugal

**Keywords:** burnout, organizational interventions, prevention, workplace

## Abstract

**Highlights:**

**Public health relevance—How does this work relate to public health issues?**

- Burnout is a widespread occupational phenomenon that affects employee health, well-being, and productivity across multiple sectors, representing a growing public health concern.
- Organizational-level interventions are critical for addressing workplace conditions that contribute to burnout, moving beyond individual-focused approaches.

**Public health significance—Why is this work of significance to public health?**

- This review identifies effective organizational strategies for preventing burnout, including psychoeducation, coaching, training programs, and third-generation therapies such as Acceptance and Commitment Therapy (ACT) and mindfulness.
- The findings highlight that most evidence comes from healthcare settings, revealing a significant gap in burnout prevention research for other professional sectors.

**Public health implications—What are the key implications or messages for practitioners, policy makers and/or researchers in public health?**

- Organizations should adopt integrated approaches combining structural workplace changes with psychoeducational and therapeutic programs to effectively prevent burnout among employees.
- Future research should explore the long-term effects of organizational interventions, extend studies to underrepresented occupational sectors, and examine how leadership engagement and institutional culture influence intervention outcomes.

**Abstract:**

Background: Burnout is an occupational phenomenon that has adverse effects on the health and work outcomes of employees. In this sense, there is greater emphasis on understanding and addressing this problem. In this regard, organizations play a significant role in preventing burnout, and organizational interventions have been shown to be more effective at reducing burnout than individual-focused interventions. This study aims to systematically review organizational interventions to prevent burnout in the workplace. Methods: A search was conducted in three databases: Scopus, Web of Science, and PubMed, reviewing studies published from 2013 to 2025. Following the PRISMA model, 11 studies meeting eligibility criteria were selected. The methodological quality of included studies was assessed using the Joanna Briggs Institute (JBI) checklist. Results: Of the eligible articles, 1669 participants were identified, predominantly female healthcare professionals. Some strategies that proved to be effective in preventing burnout in the workplace were workshops, discussion groups, psychoeducation and training programs. Concerning psychotherapeutic interventions, third-generation therapies are the most used, such as ACT or mindfulness. Conclusions: These findings suggest that organizational-level interventions, particularly those combining psychoeducational strategies with third-generation therapeutic approaches, represent promising avenues for burnout prevention in the workplace, although effectiveness varies across interventions and contexts, and the most positive effects were limited to the short term. Future research should focus on evaluating long-term outcomes, exploring understudied occupational sectors beyond healthcare, and examining the role of organizational culture and leadership in sustaining intervention effects.

## 1. Introduction

Burnout is a psychological syndrome characterized by emotional exhaustion, depersonalization, and reduced personal accomplishment, which has become a significant concern in various occupational settings worldwide [1].

This occupational phenomenon has adverse effects on the health and work-related outcomes of employees [2] and is the result of a combination of high job demands, e.g., time pressure, lack of support, stress, and work–family conflict [3], and low job resources, e.g., job control, team climate and organizational justice [3,4].

With the exposure to high job demands, employees become chronically exhausted and distance themselves psychologically from their work. In contrast, job resources can fulfill psychological needs and buffer the impact of job demands on burnout [5].

Burnout has gained increasing recognition for its detrimental impact on employee well-being and organizational outcomes [6]; therefore, there has been a greater emphasis on understanding and addressing this problem.

This syndrome should not only be highlighted due to its prevalence but also due to its outcomes, which can be on both the individual and the organizational level [4]. Regarding organizations, its implications relate to increased absenteeism, reduced organizational commitment, and increased worker intention to leave work [7], as well as lower productivity, effectiveness, and job satisfaction [4]. Engaged workers facilitate positive organizational outcomes [8].

Over the last decade, organizations have undergone continuous changes in organizational structures, which has placed increasing demands on organizational and employee adaptability and proactivity [9], which can be challenging for workers who experience burnout.

This occupational threat requires a systems approach [10] which aligns with the understanding that burnout is not solely the result of individual factors such as personal traits or coping strategies but is also influenced by organizational factors such as workload, job control, and work–life balance [6].

Effective strategies for reducing this syndrome include a combination of organizational approaches, such as implementing flexible work arrangements and promoting a supportive work culture, as well as individual-focused interventions, such as stress management training and self-care programs [11]. Some studies show that organizational interventions were more effective at reducing burnout than individual-focused interventions [12].

Organizations and their leaders play a significant role in preventing burnout and increasing job satisfaction among employees [13]. However, employees, managers and other stakeholders may have diverse conflicting agendas that may influence how they behave and react to the interventions [14].

This review aims to systematically review organizational interventions to prevent burnout in workplace. By synthesizing and analyzing a range of intervention studies across different occupational settings, this review will provide a comprehensive understanding of the current state of knowledge in this field.

There has been increasing investment in the development of organizational interventions aimed at reducing burnout. Although other reviews have addressed similar issues, this review seeks to make its contribution by focusing in detail on specific types of interventions, such as workshops, group discussions and other therapeutic programs, including third-generation therapies such as ACT and mindfulness. This approach provides a more practical and up-to-date view of how to prevent burnout in the workplace, especially in times of increasing psychological demands on workers [15].

## 2. Methods

### 2.1. Protocol Registration

This systematic review was conducted and reported in accordance with the Preferred Reporting Items for Systematic Reviews and Meta-analysis (PRISMA) 2020 guidelines [16]. The completed PRISMA checklist is provided as Supplementary Material (Table S1).

The protocol for this systematic review was registered on PROSPERO (CRD420261344451).

### 2.2. Literature Search Strategy

To carry out the systematic review, a search was conducted in the following online databases: Scopus, Web of Science, and PubMed. The first search was performed in each database in June 2023, and then it was re-run in April 2024, December 2024 and February 2026, to identify possible further studies. The keywords used in the search were: “workplace” AND “intervention” AND “organizational” AND “program” AND “burnout” AND “prevention”. Specific filters related to restrictions on publication date and language were used. Articles in English, published between 2013 and 2025, were searched.

### 2.3. Eligibility Criteria

Studies were eligible for final inclusion in the systematic review if they cumulatively met the following criteria: (i) published in a peer-reviewed journal between the years of 2013 and 2025; (ii) written in English; (iii) employing organizational interventions for managing burnout; (iv) interventions delivered in the workplace at the organizational level; and (v) using quantitative quasi-experimental or randomized controlled trial designs.

All incomplete articles or those which only presented abstracts, studies in other languages than English, studies with individual interventions, and literature reviews or systematic reviews were excluded.

### 2.4. Selection Process and Data Extraction

One reviewer screened titles and abstracts of all identified records against the eligibility criteria. Full-text articles of potentially eligible studies were then retrieved and assessed for eligibility. A second reviewer independently verified the selection, and disagreements were resolved through discussion.

**Figure 1:**
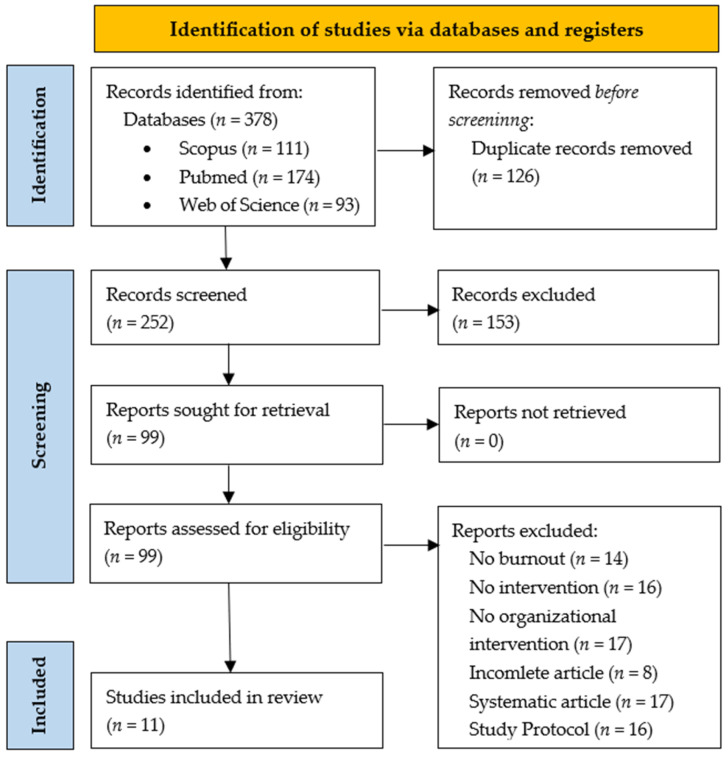
Flow chart of the study selection process.

In the initial search, articles that met the inclusion criteria, which are potentially eligible articles, were selected. Next, all duplicate articles were removed. After this, the titles and abstracts were analyzed. Taking into consideration the inclusion criteria, relevant articles were extracted and those that did not fit were excluded (Figure 1).

Data extraction was performed by one reviewer using a standardized form in Microsoft Excel and checked for accuracy by a second reviewer. The following information was summarized: name of the author(s) and year of publication, participants, study design, workplace, country, duration of sessions, duration of follow-up, instruments used, randomized studies and the study results (Table 1).

### 2.5. Study Quality

Two review authors independently assessed the quality of the eligible studies using the critical appraisal checklists from the Joanna Briggs Institute (JBI) Statistics Assessment and Review Instruments for quasi-experimental studies or randomized controlled trials (RCTs) [17]. Each item on the checklists was rated as “yes”, “no”, “unclear”, or “not applicable”. When needed, consensus was achieved through discussion with a third author. The methodological quality of the studies was evaluated based on the information provided in the articles.

**Table 1:** Summary of the studies.

Authors, Year of Publication	Study Design/ Randomized	Participants	Workplace/Country	Type of Intervention	Duration	Follow-Up	Instruments	Results
[18], 2016	Randomized controlled trial/Yes	33 primary care physicians	Providence Medical Group/USA	Mindful Medicine Curriculum (MMC)	5 months	3 months	Maslach Burnout Inventory (MBI); Mindful Attention Awareness Scale (MAAS); Brief Resilience Scale (BRS); Perceived Stress Scale–10 (PSS-10); Santa Clara Brief Compassion Scale (SCBCS); Meditation Practice Questionnaire (MPQ); Consumer Assessment of Healthcare Providers and Systems–Clinician and Group Adult Visit (CG-CAHPS)	The results showed significant improvements in the group that participated in the intervention (Mindful Medicine Curriculum): stress (*p* < 0.001); emotional exhaustion (*p* = 0.004); depersonalization (*p* = 0.01); mindfulness (*p* = 0.05).
[19], 2024	Randomized controlled trial/Yes	60 nurses	Emergency Medical Center/Iran	Mindfulness-Based Stress Reduction (MBSR) training	2 months	-	Maslach Burnout Inventory (MBI); Occupational Stress Questionnaire (OSQ)	After the intervention, there were significant differences (*p* < 0.05) in the subscales of occupational stress; the intervention group also exhibited significant differences (*p* < 0.001) in the scores of burnout subscales.
[20], 2019	Pretest–posttest control group design/Yes	77 dialysis nurses	Dialysis facilities/Germany	Dialysis-specific training program	4 months	6 weeks	Copenhagen Psychosocial Questionnaire (COPSOQ); Health-oriented Leadership (HoL); Brief COPE	A significant decrease in burnout was observed from pre-measurement (T0) to post-measurement (T1) (*p* = 0.017), with significantly different mean levels across the three time points (*p* = 0.029). However, changes between T1 and follow-up (T2) were not significant, suggesting effects were limited to the short term. Sense of community also significantly increased from T0 to T1 (*p* = 0.004).
[21], 2022	Randomized controlled trial/Yes	264 physicians	Clinics/USA	The IMPACT Program	2 years	Yes	Professional Fulfillment Index; Self-Valuation Scale; Gratitude at Work Inventory	Physicians at delayed intervention sites reported lower burnout at baseline compared to those at immediate intervention sites (*p* < 0.018). After the intervention was implemented across all sites, the change in burnout did not differ significantly between groups (*p* = 0.180). No significant differences in self-valuation were found between groups (*p* = 0.552).
[22], 2024	Randomized controlled trial/Yes	138 physicians	Hospitals/USA	Coaching groups	3 months	3 months	Maslach Burnout Inventory (MBI); Stanford Professional Fulfilment Index (PFI); Utrecht Engagement Scale-9 (UWES-9); Self-Valuation Scale; Quality of Life Scale; Impact work on Personal Relationship Scale	Mean scores for overall burnout decreased by 21.6% in the intervention group and increased by 2.5% in the control group (*p* = 0.001). The results show that individualized coaching by professionally trained peers is an effective strategy for reducing physician burnout and interpersonal disengagement while improving their professional fulfillment and work engagement.
[23], 2024	Randomized controlled trial/Yes	291 healthcare employees	Primary healthcare units/Sweden	Productivity Measurement and Enhancement System (ProMES)	17 months	6 and 12 months	Oldenburg Burnout Inventory (OLBI); AHA-questionnaire; QPS Nordic questionnaire; ERI questionnaire	Regarding burnout, ProMES did not show a significant difference in exhaustion levels over time between the intervention and the control groups (*p* = 0.477), nor was any significant difference found for recovery (*p* = 0.857) or sleeping problems (*p* = 0.634). No significant differences were observed in effort–reward imbalance (*p* = 0.903). However, a significant difference was found for job control (*p* = 0.001).
[24], 2021	Randomized controlled trial/Yes	48 clinical nurses	Hospital/Turkey	Nurse-led intervention program	4 weeks	6 months	Professional Quality of Life Scale (ProQOL-IV); GHQ-12	As a result of the intervention applied in this study, it was determined that there was no difference between the groups in terms of burnout (pretest: *p* = 0.777; posttest 1: *p* = 0.145; posttest 2: *p* = 0.392).
[25], 2022	Randomized controlled trial/Yes	42 nurses	Hospital/Republic of the Marshall Islands	Personalized music intervention	5 weeks	-	Maslach Burnout Inventory (MBI)	After adjusting for baseline differences, the intervention group showed significantly lower emotional exhaustion compared to the control group (*p* = 0.005). However, no significant differences were found for the other two MBI subscales: depersonalization (*p* = 0.30) and personal accomplishment (*p* = 0.06). No significant differences were observed in psychological distress (depression, anxiety, and stress) between groups. Sleep quality improved significantly in the intervention group (*p* = 0.004).
[26], 2018	One-group pretest-posttest design/No	67 employees	Healthcare Institutions/-	Burnout Prevention Team	10 sessions	3 and 9–15 months	Maslach Burnout Inventory-General Survey (MBI-GS); Areas of Worklife Scale (AWS); Well-being Index (WHO 5)	Participants in the BPT significantly increased their knowledge about work-related risk factors contributing to burnout (*p* < 0.01).
[27], 2022	Randomized controlled trial/Yes	146 healthcare staff	National Health Service primary care/England	Acceptance and Commitment Therapy (ACT)	1 year	14 weeks	Shirom–Melamed Burnout Measure (SMBM); General Health Questionnaire (GHQ-12); Affective Rumination Scale; Perseverative Cognition Scale; Short-Form Five Facet Mindfulness Questionnaire; Valuing Questionnaire; Self-Compassion Scale–short-form	The results advance an ACT-based workplace as an effective stress management intervention for healthcare staff. Its effects are significant from baseline to mid-intervention (*p* = 0.004) and from baseline to post-intervention (*p* = 0.004), but the interaction was not significant from baseline to follow-up (*p* = 0.22). The cognitive weariness (burnout sub-component) significantly declined from baseline to mid-intervention (*p* = 0.004), post-intervention (*p* = 0.04) and follow-up (*p* = 0.02).
[28], 2022	Randomized controlled trial/Yes	456 industries employees	Industries/-	Web-based Psychoeducation Intervention program	9 months	3 months	Maslach Burnout Inventory (MBI); Depression, Anxiety, and Stress scale (DASS); European Quality of Life-5 Dimensions (EQ-5D-5L); Australian National Mental Health Literacy and Stigma Survey.	Significant differences between intervention and control groups were found on all outcome measures except the self-rated quality of life. The intervention group displayed a significant reduction in the weighted mean score on the stress scale (*p* = 0.015) and an increase in the Burnout—Professional Accomplishment domain of the MBI (*p* = 0.035).

## 3. Results

### 3.1. Study Selection

After initial searches, 378 potentially eligible studies were identified: 111 in Scopus, 93 in Web of Science and 174 in PubMed. After the removal of duplicates (*n* = 126), 252 possibly eligible studies remained. At the end of the selection of titles and abstracts, 153 articles were excluded, leaving 99 studies. Of these, 88 were excluded, leaving 11 studies that met all the selection criteria, according to the flowchart (Figure 1).

Of the 99 full-text articles assessed for eligibility, 88 were excluded for the following reasons: not an organizational-level intervention (*n* = 17), not an interventional study (*n* = 16), no burnout outcome measured (*n* = 14), study incomplete (*n* = 8), systematic review studies (*n* = 17) and study protocols (*n* = 16).

### 3.2. Study Characteristics and Participants

Eleven articles were included in the present review, whose data are systematized in Table 1, which shows that all selected studies present intervention programs in the workplace.

The majority of participants in the analyzed studies are women. There are two studies [18,19] that do not refer to the gender of the participants, encompassing 93 study subjects. In the total of 1669 subjects, there are 1088 women, 439 men, and 142 undefined/unidentified, because in one study two participants did not specify their gender.

The mean age of the participants is 40.68 years old, with the minimum being 34.72 years and the maximum being 45.83 years. There are three studies that refer to the ages of the participants in age ranges however [20,21,22].

One of the selected studies refers the number of years that the employee has worked in the organization [23]. There are 136 participants who have worked in the organization for at least 5 years, 52 participants who have worked between 6 and 10 years in the organization and 61 who have worked in the organization for more than 10 years. In four of the selected studies, there is mention of the number of years the employees have been working in the profession [20,22,23,24]; however, in one of them this reference is an average (12.3 y) [22]. There are 188 participants who have been working in the profession for at least 10 years, while there are 151 who have been working in the profession for 10 years or more. There is a study that refers to the duration of employment, not in years, but in percentage, with the periods between 6 and 10 years and between 16 and 20 years having the highest percentage of employees [20].

From the total number of articles (*n* = 11), it is possible to show that the majority of participants were health professionals, namely nurses [19,20,21,23,24,25]. In addition to this, there are also studies with physicians [18,21,22,23], physiotherapists, medical secretaries, midwifes, laboratory technicians, assistant nurses, counselors, managers/assistant managers, dietitians [23], physician assistants [21] and nursing home employees [26]. There is one study that also involves health professionals/healthcare professionals but does not specify the participants’ jobs [27]. It should be noted that there is a study involving industrial workers, which is the only study to include participants from a profession outside the healthcare sector [28].

The years of publications of the selected studies occur from 2016 to 2024. The year with the highest number of published articles (*n* = 4) is 2022 [Min = 0; 2017, 2020, 2021; Max = 4; year 2022; SD = 1.30].

The selected articles present diversity regarding the country in which the intervention program was implemented: Sweden [23], USA [18,21,22], Germany [20], Republic of the Marshall Islands [25], Turkey [24], Iran [19] and England [27]. Two articles do not identify in which country the intervention was implemented.

The studies were developed in various workplaces, such as primary healthcare units/Healthcare Centers/National Health Service primary care/Healthcare Institutions [23,26,27], Medical Centers/Hospitals/Clinics [18,19,21,22,24,25], dialysis facilities [20] and industries [28].

### 3.3. Outcome Measures and Follow-Up

The instruments administered across the selected studies are heterogeneous. Regarding burnout assessment, the most frequently used instrument was the Maslach Burnout Inventory (MBI), applied in six studies [18,19,22,25,26,28]. Other instruments specifically used to measure burnout included the Oldenburg Burnout Inventory (OLBI) [23], the Shirom–Melamed Burnout Measure (SMBM) [27], and the Professional Quality of Life Scale (ProQOL-IV), which includes a burnout subscale [24,27]. The Professional Fulfillment Index (PFI), which features a burnout component, was used in two studies [21,22], and the Copenhagen Psychosocial Questionnaire (COPSOQ), which also contains a burnout dimension, was applied in one study [20].

In addition to burnout-specific measures, several instruments were used to assess related variables. Stress was assessed using the Perceived Stress Scale–10 (PSS-10) [18], the Depression, Anxiety, and Stress Scale (DASS) [28], and the Occupational Stress Questionnaire (OSQ) [19]. General psychological well-being was measured through the General Health Questionnaire (GHQ-12) [24,27], the Well-being Index (WHO-5) [26], and the European Quality of Life–5 Dimensions (EQ-5D-5L) [28]. Mindfulness and related constructs were assessed using the Mindful Attention Awareness Scale (MAAS), the Five Facet Mindfulness Questionnaire, the Valuing Questionnaire, and the Self-Compassion Scale [18,27]. Work-related variables were measured through the Areas of Worklife Scale (AWS) [26], the AHA-questionnaire, the QPS Nordic questionnaire, and the ERI questionnaire [23], the Utrecht Work Engagement Scale-9 (UWES-9) and the Quality of Life Scale [22], the Self-Valuation Scale [21,22], the Gratitude at Work Inventory [21], the Health-oriented Leadership (HoL) questionnaire and the Brief COPE [20], the Impact of Work on Personal Relationship Scale [22], the Brief Resilience Scale (BRS) and the Santa Clara Brief Compassion Scale (SCBCS) [18], the Meditation Practice Questionnaire (MPQ) [18], the Consumer Assessment of Healthcare Providers and Systems–Clinician and Group Adult Visit (CG-CAHPS) [18], the Affective Rumination Scale and the Perseverative Cognition Scale [27], and the Australian National Mental Health Literacy and Stigma Survey [28].

Of note, follow-up occurs in nine studies [18,20,21,22,23,24,26,27,28]. The follow-up occurs at various time points: 6 and 12 months [23] and 3 and 9–15 months [26]. Studies with a follow-up of 6 months [24], 3 months [18,22,28], 14 weeks [27] and 6 weeks [20] after the intervention were found. One of the selected articles presents follow up but does not specify how long after the intervention [21]. Two articles do not present follow-up.

Most of the studies are randomized; ten studies follow this type of method [18,19,20,21,22,23,24,25,27,28].

### 3.4. Intervention Characteristics

The duration of the interventions varied considerably across studies, ranging from 4 weeks [24] to 24 months [23]. Specifically, the shortest interventions lasted 4 weeks [24] and 5 weeks [25], while others extended to 2 months [19], 3 months [22], 4 months [20], 5 months [18], 9 months [28], 12 months [27], and 17 months [23].

The types of intervention found in the selected articles are workshops, e.g., about motivation [23] and mindfulness [18,19,21], psychoeducational programs [28], discussion groups/group sessions [26], coaching [22] music intervention [25] and training programs [20]. There also are programs based on a cognitive–behavioral approach [24,27].

### 3.5. Intervention Effectiveness

Regarding the effectiveness of the interventions, the study by Arapovic-Johansson et al. [23] implemented the Productivity Measurement and Enhancement System (ProMES) and presented three research questions; however, only one was related to burnout, since it was assessed whether ProMES can reduce exhaustion levels (measured through the Olderburg Burnout Inventory), concluding that it did not show significant differences (*p* = 0.857). Furthermore it also did not show significant differences in exhaustion over time between the control and experimental groups (*p* = 0.477).

However, the Burnout Prevention Team (BPT), an organizational-level program in which a multidisciplinary team identifies workplace-specific burnout risk factors and develops tailored prevention strategies, presented good results in relation to its effectiveness in preventing burnout, given that BPT members reported knowledge gains confirmed by pre–post comparison of the quiz (*p* < 0.01) [26].

One study assessed the effects of the intervention across three time points: pre-measurement (T0), post-measurement (T1), and follow-up (T2). The results showed significantly different mean levels between the three measurements for burnout (*p* = 0.029). Specifically, a significant decrease in burnout was observed from pre-measurement (T0) to post-measurement (T1) (*p* = 0.017). However, the changes between post-measurement (T1) and follow-up (T2) were not statistically significant, suggesting that the positive effects of the intervention were limited to the short term [20].

The participants of the Web-based Psychoeducation Intervention displayed a significant reduction in the weighted mean score on the stress scale (*p* = 0.015) and an increase in the Burnout—Professional Accomplishment domain of the MBI (*p* = 0.035). The other domains of MBI, depersonalization and emotional exhaustion, did not show significant differences [28].

In addition, participants who received personalized music intervention experienced less emotional exhaustion (*p* = 0.005), which means the personalized music intervention can be used as an adjuvant approach to reduce emotional exhaustion, a domain of MBI [25].

As a result of the intervention applied in another study, which addressed a program based on a cognitive–behavioral approach, it was determined that there was no difference between the groups in terms of burnout (pretest: *p* = 0.777; posttest 1: *p* = 0.145; posttest 2: *p* = 0.392) [24].

Nevertheless, workplace Acceptance and Commitment Therapy (ACT) proved to be an effective stress management intervention for healthcare staff. It was significant from baseline to mid-intervention (*p* = 0.004) and from baseline to post-intervention (*p* = 0.004) but the interaction was not significant from baseline to follow-up (*p* = 0.22). The cognitive weariness (burnout sub-component) significantly declined from baseline to mid-intervention (*p* = 0.004), post-intervention (*p =* 0.04) and follow-up (*p* = 0.02) [27].

The IMPACT Program, a controlled organizational intervention that uses influential peer volunteers to lead wellness-oriented workshops, used a randomized design in which clinical sites were assigned to receive the intervention either immediately or after a delay. At baseline, physicians at delayed intervention sites reported lower levels of burnout compared to those at immediate intervention sites (*p* < 0.018). However, after the implementation of the intervention, the change in burnout did not differ significantly between the two groups (*p* = 0.180), indicating that the program did not produce a measurable differential effect on burnout reduction [21].

Individualized coaching by professionally trained peers also proved to be an effective strategy for reducing physician burnout and interpersonal disengagement while improving their professional fulfillment and work engagement. Mean scores for overall burnout decreased by 21.6% in the intervention group and increased by 2.5% in the control group (*p* = 0.001) [22].

The participants of the intervention group of Mindful Medicine Curriculum [18], a brief mindfulness-based program, showed significant improvements in emotional exhaustion (*p* = 0.004) and depersonalization (*p* = 0.01). These results suggest that the mindfulness-based intervention effectively reduced aspects of burnout among the participating physicians. There was no significant improvement in resilience (*p* = 0.14), compassion (*p* = 0.66), or personal achievement (*p* = 0.06) for the intervention group, indicating that while some aspects of burnout improved, others did not show significant changes.

The results regarding burnout in the Mindfulness-Based Stress Reduction (MBSR) training [19] indicated significant differences between the intervention group and the control group. The intervention group showed statistically significant improvements in all three dimensions of burnout post-intervention: emotional exhaustion (*p* < 0.001) and depersonalization (*p* < 0.001) and reduced personal accomplishment (*p* < 0.001).

## 4. Discussion

The present systematic review seeks to analyze 11 articles, published between 2016 and 2024, which aimed at sharing burnout intervention with professionals across different occupational settings in their workplace, with a peak of four articles published in 2022. This concentration may not be coincidental, as it likely reflects the heightened global awareness of occupational burnout triggered by the COVID-19 pandemic, which placed unprecedented demands on healthcare systems.

Through analysis of selected articles, it is possible to note that there are intervention programs focused on team meetings and workshops about motivation, goals, contingencies, etc. This increases awareness about burnout and its implications [1], helping to prevent it.

A psychoeducation intervention program targeting the workplace is, in the short term, effective in reducing workplace burnout and stress and promoting mental health literacy at the workplace. The Web-based Psychoeducation Intervention displayed a significant reduction in the weighted mean score on the stress scale and an increase in professional accomplishment (Burnout), a domain of MBI [28]. One study corroborates that psychoeducation significantly reduces burnout by developing coping strategies and ensuring therapeutic collaboration [29]. Another study conducted a training program designed to address stress management and promote health among employees, with a special focus on the role of supervisors, which also proved to be effective in reducing participants’ burnout levels, at least in the short term [20]. Also, the participants in the Burnout Prevention Team (BPT) significantly increased their knowledge about work-related risk factors contributing to burnout; therefore, the BPT program can be proposed as a good and effective example for a tailored approach that considers the organizational conditions and challenges of transferring intervention results into the daily work practices of each institution [26].

Providing workers with knowledge about the concept of burnout and the factors that trigger it, like psychoeducation, has proven to be a good way to develop successful intervention solutions [26], although both the psychoeducation intervention and the training program have only shown effectiveness in the short term.

It is also possible to verify that implementing a personalized music intervention, which also may be an effective remedy for reducing emotional exhaustion [25], since musical interventions significantly reduce stress, can help prevent the risk of burnout [30].

Besides these, individualized coaching by professionally trained peers is an effective strategy for reducing physician burnout and interpersonal disengagement while improving their professional fulfillment and work engagement [23]. In fact, coaching technologies can effectively prevent professional burnout by increasing resilience and stress resilience [31], since they allow people to deal with burnout by increasing the internal locus of control, improving self-awareness and aligning personal values with professional duties [32].

Concerning psychotherapeutic interventions, workplace Acceptance and Commitment Therapy (ACT) showed positive effects on psychological stress and cognitive fatigue, as well as significant improvements in reducing work-related worries [27]. Cognitive weariness (burnout sub-component) significantly declined with this intervention. Moreover, a wellness intervention based on positive psychology workshops led to favorable changes in worker satisfaction and gratitude, which improved occupational well-being [21], which in turn contributes to the prevention of burnout since a positive work environment is an effective strategy in preventing this syndrome [33].

Through the analysis of the selected articles, it is possible to see that not all implemented interventions show favorable results regarding the prevention of burnout in the workplace. In other words, it was not possible to prove the positive impact of the interventions in some aspects, such as in relation to the reduction in perceived stress, exhaustion, sleep difficulties, and workload levels [23]. Even though this study does not show significant differences, the results show differential effects of ProMES on exhausted/non-exhausted employees, which is an intervention that could have a preventive effect on healthy individuals, but this effect could cease when they were exhausted. Besides this, a program based on a cognitive–behavioral approach proved not to be effective in preventing burnout; however, the results showed that people who participated in this intervention program reduced their level of distress [24]. Similarly, a psychoeducational training program for teachers, which included self-awareness exercises, autogenic training techniques, and conflict management strategies, did not yield significant reductions in burnout levels, psychological immune competence, or coping strategies, possibly due to the small sample size and the short follow-up period [34]. These findings suggest that the effectiveness of psychoeducational interventions may depend on factors such as the length and intensity of the program, the sample size, and participants’ motivation and commitment.

Preventive organizational interventions are not meant to be therapeutic [23]; i.e., the main objective of preventive interventions in organizations is to prevent the occurrence of problems and they are not aimed at treating existing problems. Preventive interventions in organizations mainly consist of skills training and a combination of individual-, group- and organizational-level interventions, leading to positive work and mental health outcomes [35].

To reduce burnout, it is suggested that, in conjunction with organizational interventions, individual approaches be considered, such as problem-solving therapies, especially for people who already show signs of exhaustion [23]. Combinatorial strategies combining individual and organizational interventions can effectively mitigate burnout, improving job satisfaction [36].

Some strategies that proved to be effective in preventing burnout in workplace were coaching [22], psychoeducation [26], training programs [20] and music intervention [25]. Concerning psychotherapeutic interventions, third-generation therapies are the most used, such as mindfulness or Acceptance and Commitment Therapy (ACT) [18,19,21,27].

Through a review of selected articles, it is possible to mention that although interventions vary in their approach, there is no single solution and the effectiveness of interventions depends on the specific context and participant characteristics.

An important finding of this review is the notable concentration of studies on healthcare professionals. Of the 11 selected articles, 10 focused exclusively on healthcare workers, including nurses, physicians, and other clinical staff, with only one study [27] involving participants from an industrial setting. This predominance likely reflects the historically higher recognition of burnout as a critical issue in healthcare, where emotional demands, exposure to suffering, and high-stakes decision-making create particularly challenging work environments.

A limitation of this review is the emphasis on preventative measures, which may not adequately address the needs of workers already experiencing burnout. Moreover, the lack of focus on long-term organizational changes limits our understanding of their wider impact. In addition, there is a clear majority of studies in the healthcare sector, leaving other fields largely underexplored. These gaps provide an idea for future research, allowing us to guide efforts that integrate preventive approaches, as well as address structural issues in the workplace, to achieve more durable and effective outcomes.

## 5. Conclusions

This systematic review provides a comprehensive overview of interventions aimed at preventing burnout in various occupational contexts. The findings reinforce the relevance of a multifactorial approach that considers both individual and organizational aspects in the formulation of effective coping strategies.

Although numerous systematic reviews on burnout interventions have been published in recent years, most have focused broadly on combined individual and organizational strategies or have examined specific populations without differentiating between intervention levels. This review adds to the existing literature by focusing exclusively on organizational-level interventions, which have been shown to be more effective than individual-focused approaches in addressing the structural and contextual factors that contribute to burnout.

Evidence shows that interventions such as psychoeducation programs, individualized coaching, leadership and wellness training, third-generation therapies (such as ACT), and even musical interventions have shown positive results in reducing stress and increasing professional engagement, although in many cases these effects have been limited to the short term. These strategies contribute to strengthening mental health at work by promoting knowledge, emotional self-regulation, resilience, and greater alignment between personal values and professional goals.

However, the effectiveness of the interventions was not uniform across studies. In some cases, no significant improvements were observed in variables such as exhaustion, perceived stress, and sleep difficulties, indicating that burnout prevention requires more than one-off interventions. It is necessary to recognize that preventive interventions alone are not therapeutic in nature and may have limited effects on workers who are already in the advanced stages of the syndrome.

Another important point concerns the contextualized nature of interventions. The success of strategies depends directly on organizational conditions, leadership involvement, and institutional culture. In addition, the predominance of studies with healthcare professionals reveals an important gap in the literature, leaving aside other professional categories that are equally vulnerable to burnout.

In this sense, the importance of integrated strategies that combine individual actions with structural and sustainable organizational changes stands out. Promoting psychologically safe work environments, with clear roles, balanced workloads, and emotional support, is as essential as empowering workers to deal with the emotional challenges of their job.

The gaps identified in this review point to clear directions for future research, such as further exploration of understudied sectors, assessment of long-term effects, and analysis of interventions aimed at organizational restructuring. Such investigations are fundamental to the development of more inclusive, durable, and effective approaches to prevent burnout.

Finally, preventing burnout requires an ongoing commitment to mental health in the workplace, an effort that must be shared by professionals, managers, and institutions. Effective interventions not only benefit individual well-being but also contribute to building healthier, more resilient, and sustainable work environments.

## Data Availability

The data that support the findings of this study are available from the corresponding author upon reasonable request.

## Supplementary Materials

The following supporting information can be downloaded at: https://www.mdpi.com/article/10.3390/ijerph23050556/s1, Table S1: PRISMA_2020_checklist_S1.

## Abbreviations

The following abbreviations are used in this manuscript:

ACT	Acceptance and Commitment Therapy
AHA	Work and Health in Everyday Life Questionnaire
AWS	Areas of Worklife Scale
BPT	Burnout Prevention Team
BRS	Brief Resilience Scale
CG-CAHPS	Consumer Assessment of Healthcare Providers and Systems—Clinician and Group Adult Visit
COPSOQ	Copenhagen Psychosocial Questionnaire
DASS	Depression, Anxiety, and Stress Scale
EQ-5D-5L	European Quality of Life—5 Dimensions
ERI	Effort–Reward Imbalance
GHQ-12	General Health Questionnaire-12
JBI	Joanna Briggs Institute
MAAS	Mindful Attention Awareness Scale
MBI	Maslach Burnout Inventory
MBI-GS	Maslach Burnout Inventory—General Survey
MBSR	Mindfulness-Based Stress Reduction
MMC	Mindful Medicine Curriculum
MPQ	Meditation Practice Questionnaire
OLBI	Oldenburg Burnout Inventory
OSQ	Occupational Stress Questionnaire
PFI	Professional Fulfillment Index
PRISMA	Preferred Reporting Items for Systematic Reviews and Meta-Analyses
ProMES	Productivity Measurement and Enhancement System
ProQOL	Professional Quality of Life Scale
PROSPERO	International Prospective Register of Systematic Reviews
PSS-10	Perceived Stress Scale-10
QPS	General Questionnaire for Psychological and Social Factors at Work
RCT	Randomized Controlled Trial
SCBCS	Santa Clara Brief Compassion Scale
SD	Standard Deviation
SMBM	Shirom–Melamed Burnout Measure
UWES-9	Utrecht Work Engagement Scale-9
WHO-5	World Health Organization Well-Being Index 5
